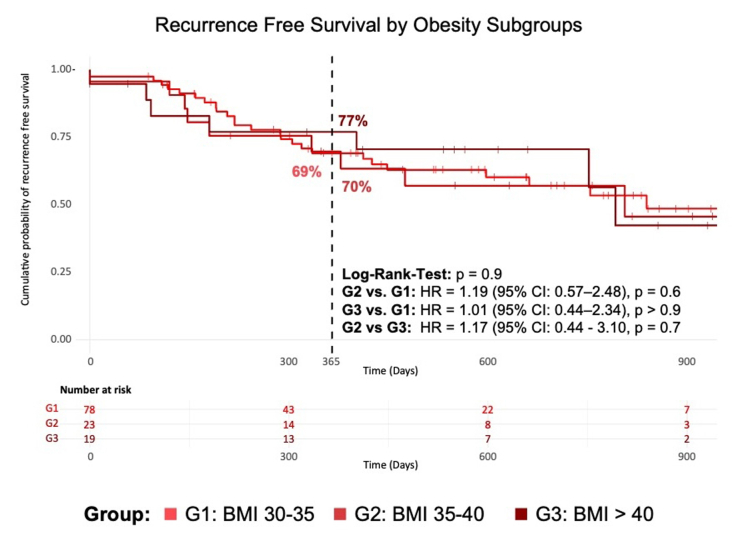# De novo pulmonary vein isolation in obese vs nonobese patients under deep sedation: Does obesity increase procedure complexity?

**DOI:** 10.1016/j.hroo.2025.06.024

**Published:** 2025-07-08

**Authors:** Vera Maslova, Marie Ahrens, Ole Rosenthal, Theodor Bau, Peter Magerfleisch, Fabian Moser, Adrian Zaman, Mohammed Saad, Martina Spehlmann, Derk Frank, Evgeny Lian

**Affiliations:** 1 Department of Internal Medicine III, Cardiology and Angiology, University Hospital Schleswig-Holstein, Kiel, Germany; 2 German Centre for Cardiovascular Research, Partner Site North, Kiel, Germany

**Keywords:** Atrial fibrillation, Catheter ablation, Deep sedation, Obesity, Cryoballoon ablation, Pulsed field ablation, Propofol

## Abstract

**Background:**

Pulmonary vein isolation (PVI) in obese patients under deep sedation (DS) is anticipated to be more complex owing to challenging airway and hemodynamic management and dose adjustment of sedation drugs.

**Objective:**

This study aimed to compare the complexity of de novo PVI in obese vs nonobese patients, with a particular focus on periprocedural sedation.

**Methods:**

All patients undergoing de novo PVI under DS between January 2022 and January 2024 in our center were prospectively included. Data on detailed monitoring of respiratory and hemodynamic parameters during the procedure were collected. Two groups were defined (group 1, body mass index [BMI] of ≥30 kg/m^2^; group 2, BMI of <30 kg/m^2^) and compared in terms of DS tolerance, safety, and procedural success.

**Results:**

Overall, 381 patients were included (61% male, median age 69 years); 120 were assigned to group 1 (BMI of 33 kg/m^2^ [32–38]) and 261 to group 2 (BMI of 25 kg/m^2^ [23–27]); 69% underwent cryoballoon ablation, 22% radiofrequency ablation, and 9% pulsed field ablation. The incidence of hypotension did not differ between groups. Hypoxic episodes were more frequent in group 1 (4 vs 2, *P* < .05), but none required mechanical ventilation. In multivariate analysis, obesity alone was not an independent risk factor for hypoxia or hypotension. Procedural duration, left atrial (LA) dwell time, and radiation dose were significantly higher in group 1. Overall complication rate was 3.4%, with no difference between groups. The 1-year success rate was comparable (71% vs 63%, *P* = .13). Subgroup analysis for persistent atrial fibrillation revealed a higher 1-year success rate (70% vs 57%, *P* = .048) for group 1.

**Conclusion:**

Obesity was not an independent risk factor for periprocedural hypoxia or hypotension and did not affect safety or long-term success. Obesity alone should not be considered a reason to exclude patients from undergoing PVI under DS.

## Graphical abstract

**Figure:**
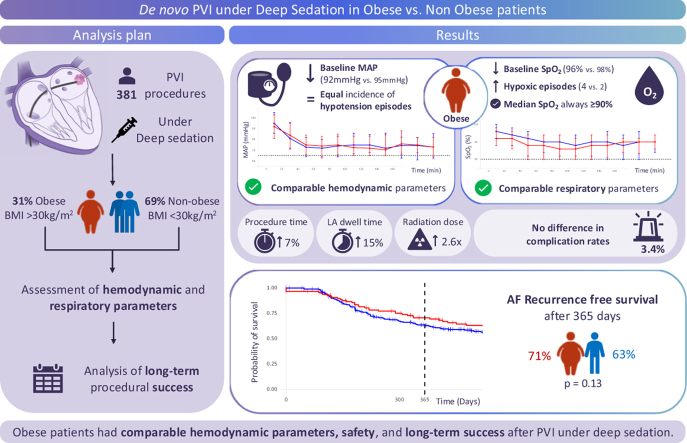

**Key Findings**

▪ Obese patients tolerated deep sedation hemodynamically well with no significant differences in the number of hypotension episodes, mean arterial pressure (MAP) levels, or degree of MAP reduction compared with nonobese patients.
▪ Although obese patients experienced more hypoxic episodes than nonobese patients, these were managed without complications. The median oxygen saturation level and first quartile remained above 90% throughout the entire procedure in both groups.
▪ In multivariate analysis, obesity alone was neither a risk factor for hypoxia nor hypotension.
▪ Although the procedure duration and left atrial dwell time were longer in obese patients and they received a higher fluoroscopy dose, the safety profile remained comparable between the groups.
▪ Recurrence-free survival did not differ between obese and nonobese patients; however, among patients with persistent atrial fibrillation, the recurrence rate was lower in the obese group.

## Introduction

Atrial fibrillation (AF) is one of the most common arrhythmias and is associated with increased morbidity and mortality.^1^ The global prevalence of AF is rising rapidly owing to population ageing, a growing burden of comorbidities, and earlier detection.^2^^,^^3^ Pulmonary vein isolation (PVI) is a cornerstone in AF treatment and can be performed as radiofrequency (RF), cryoballoon (CB), or pulsed field ablation (PFA), which are noninferior to each other in terms of efficacy.^4^, ^5^, ^6^, ^7^ Obesity is a known risk factor for AF, the prevalence of which increases dramatically in Western countries.^8^, ^9^, ^10^ Hence, the number of overweight patients requiring catheter ablation (CA) is also rising.

Given that data on the impact of body mass index (BMI) on long-term procedural success remain controversial, the PVI procedure itself can be challenging in obese patients owing to hemodynamic and respiratory compromise, limited fluoroscopic visibility of cardiac structures, and enlarged atria. The anticipated procedure complexity may lead to the refusal to perform PVI in obese patients. Deep sedation (DS) is one of the most commonly used sedation techniques for PVI worldwide.^11^ However, data comparing hemodynamic and respiratory stability between obese and nonobese patients undergoing PVI under DS are currently lacking. In this study, we sought to compare the complexity of de novo PVI procedures in obese vs nonobese patients, with a particular focus on periprocedural sedation. We report on periprocedural hemodynamic and respiratory parameters, safety, and both acute and long-term procedural success.

## Methods

### Patient population

The study was performed based on our prospective institutional registry database. We included all patients who underwent de novo PVI owing to symptomatic paroxysmal or persistent AF between January 2022 and January 2024 in our hospital. Procedures were performed with either RF energy (with CARTO System, Biosense Webster, Irvine, CA; EnSite System, Abbott, St. Paul, MN; or Rhythmia System, Boston Scientific, Cambridge, MA) or single-shot devices: CB ablation (Arctic Front, Medtronic, Minneapolis, MN, or POLARx, Boston Scientific) or PFA (FARAPULSE, Boston Scientific). We excluded patients who underwent additional substrate modification in the LA beyond PVI. Based on BMI, the study population was divided into 2 groups: group 1, BMI of ≥30 kg/m^2^ (obese), and group 2, BMI of <30 kg/m^2^ (nonobese). A written informed consent was obtained from all patients. The study protocol was approved by the local ethical committee. The study complied with the Declaration of Helsinki.

### Ablation procedure and sedation protocol

On the day of ablation, patients skipped the morning dose of novel oral anticoagulants. Those on vitamin K antagonists have kept the international normalized ratio between 2 and 3. The choice of ablation technique—RF ablation, CB CA, or PFA—was based on the operator’s preference.

DS was defined as a drug-induced depression of consciousness during which patients cannot be easily aroused but respond purposefully after repeated or painful stimulation, according to the definition of the American Society of Anesthesiologists.^12^ During the study period, no patients underwent PVI under general anesthesia (GA). DS was administered and monitored by nurses under the direct supervision of the electrophysiologist. All procedures were performed under DS with propofol and fentanyl; in some cases midazolam was added, following a standardized DS protocol at our institution: induction with a bolus of propofol (0.8–1 mg/kg of body weight) and fentanyl (0.05 mg), followed by continuous propofol infusion via a syringe pump. Additional doses of fentanyl or midazolam were administered during the procedure if necessary. The propofol infusion rate was adjusted based on hemodynamic parameters, such as blood pressure (BP), peripheral oxygen saturation (SpO_2_), and level of consciousness. BP was measured noninvasively every 5 minutes, whereas SpO_2_ was continuously monitored using a finger pulse oximeter throughout the procedure. For all patients, BP and SpO_2_ levels were documented every 10 minutes in the Mac-Lab system (GE HealthCare, Chicago, IL). An oropharyngeal airway tube was introduced for every patient. After the procedure, the measurements were exported from the Mac-Lab system for further analysis. Hypoxia was defined as SpO_2_ of <90%, and hypotension as mean arterial pressure (MAP) of <65 mm Hg.

Periprocedural anticoagulation was achieved by unfractionated heparin at a dose of 100 IU per kg of body weight. For RF ablation, 2 transseptal punctures (TSPs) were performed, whereas for single-shot device procedures 1 puncture was used. Angiographic visualization of pulmonary veins with contrast medium was performed irrespective of ablation technique.

After the procedure, patients were routinely monitored overnight and discharged the next day. After CA, an antiarrhythmic medication was prescribed for 3 months during the blanking, while beta-blockers were continued.

### Follow-up

Recurrence was defined as AF or atrial tachycardia, occurring after a 3-month blanking period, lasting longer than 30 seconds, confirmed by a 12-lead electrocardiogram (ECG) or Holter ECG. Follow-up visits at 3, 6, and 12 months, including a 24-hour Holter ECG, were performed either at our institution or at a referral center.

### Statistical analysis

Continuous variables were reported as the median and interquartile range (25%–75%) where appropriate. Categorical variables were reported as percentages. Continuous variables between the 2 groups were compared using *t* test for uniform distribution and the Mann-Whitney U test for nonuniform distribution. χ^2^ test was used for comparison of categorical variables. A *P* value of <.05 (2 tailed) was considered statistically significant. Survival analyses were performed using the Kaplan-Meier method. Statistical calculations were conducted using the R statistical language (R Foundation for Statistical Computing, Vienna, Austria).^13^

## Results

### Study population

**Figure 1:**
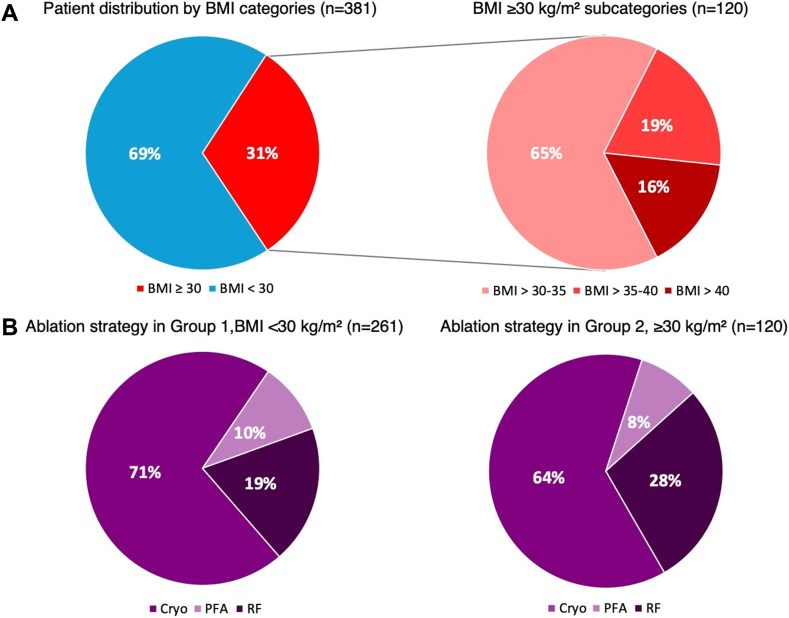
**A:** Patient distribution according to BMI. **B:** Ablation strategy in different BMI groups. BMI = body mass index; Cryo = cryoballoon ablation; PFA = pulsed field ablation; RF = radiofrequency.

A total of 381 patients who underwent de novo PVI between January 2022 and January 2024 were included: 233 male (61%), with a median age of 69 years (61–75). In 209 patients (55%) AF was paroxysmal, and in 172 (45%) persistent. Among the 381 enrolled patients, 120 (31%) had a BMI of ≥30 kg/$m^{2}$ (group 1, obese) and 261 (69%) had a BMI of <30 kg/$m^{2}$ (group 2, nonobese). In the obese group, 78 patients (65%) had a BMI of 30–35 kg/$m^{2}$, 23 (19%) 35–40 kg/$m^{2}$, and 19 (16%) >40 kg/$m^{2}$ (Figure 1A).

Baseline characteristics in the overall cohort and comparisons between the 2 groups are presented in Table 1. Obese patients were younger (64 vs 70 years, *P* < .001), had a higher prevalence of persistent AF (55% vs 41%, *P* = .008), and had more comorbidities such as hypertension (90% vs 72%, *P* < .001), diabetes mellitus (23% vs 12%, *P* = .004), chronic lung disease (18% vs 7%, *P* < .001), and higher baseline glucose levels (105 vs 99 mg/dL, *P* < .001). The LA diameter was larger in obese patients (45 vs 41 mm, *P* = .02), whereas LA volume index was similar in both groups. There were no statistically significant differences in left ventricular ejection fraction (LVEF), coronary artery disease, and renal insufficiency between groups. Obese patients were more often anticoagulated with apixaban (53% vs 39%, *P* = .007). There were no differences in the CHA_2_DS_2_-VA score and other anticoagulation and antiarrhythmic drugs between groups.

**Table 1:** Patient baseline characteristics according to BMI

Patient baseline characteristics by BMI	Total (n = 381)	BMI of ≥30 kg/m^2^ (n = 120)	BMI of <30 kg/m^2^ (n = 261)	*P* value
Demographics				
Age, y	69 [61–75]	64 [59–72]	70 [62–77]	<.001
Men, %	233 (61)	75 (63)	158 (61)	.71
Type of AF, %				
Paroxysmal AF	209 (55)	54 (45)	155 (59)	.009
Time after initial diagnosis				
<6 mo	99 (26)	29 (24)	70 (27)	.58
≥6 mo	185 (49)	65 (54)	120 (46)	.14
CHA_2_DS_2_-VA score				
Median, IQR	3 [2–4]	3 [2–3]	3 [2–4]	.28
≥2, %	296 (78)	91 (76)	205 (79)	.55
Comorbid conditions				
BMI, kg/m^2^	27 [24–31]	33 [32–38]	25 [23–27]	0∗
Hypertension, %	295 (77)	108 (90)	187 (72)	<.001∗
Diabetes mellitus, %	59 (15)	28 (23)	31 (12)	.004∗
CAD, %	157 (41)	45 (38)	112 (43)	.32
Impaired kidney function (GFR of <60), %	108 (28)	34 (28)	74 (28)	1
s/p stroke/TIA, %	31 (8)	5 (4)	26 (10)	.06
Respiratory disease, %	40 (11)	21 (18)	19 (7)	.004∗
Smoker, %	124 (33)	44 (37)	80 (31)	.24
Alcohol abuse, %	15 (4)	5 (4)	10 (4)	1
Implanted device, %	39 (10)	9 (8)	30 (11)	.27
Laboratory values				
LDL, mmol/L	2 [2–3]	3 [2–3]	2 [2–3]	.27
NT-proBNP, ng/L	629 [244–1583]	585 [270–1159]	789 [243–1939]	.11
Glucose, mg/dL	101 [91–113]	105 [93–126]	99 [90–109]	<.001∗
Echocardiographic data				
LVEF, %	55 [45–60]	55 [45–60]	60 [50–60]	.12
LVEF of <35%, %	40 (11)	11 (9)	29 (11)	.57
LA diameter, mm	43 [38–47]	45 [39–48]	41 [38–47]	.02∗
LAVI, mL/m^2^	39 [30–47]	38 [27–44]	39 [30–47]	.25
AAD therapy, %				
Beta-blockers	354 (93)	109 (91)	245 (94)	.28
Amiodarone	30 (8)	12 (10)	18 (7)	.30
Class I AR	18 (5)	6 (5)	12 (5)	.86
OAC therapy, %				
NOACs	360 (95)	114 (95)	246 (94)	.77
Apixaban	165 (43)	64 (53)	101 (39)	.007∗
Rivaroxaban	152 (40)	41 (34)	111 (43)	.12
Edoxaban	39 (10)	9 (8)	30 (11)	.23
Dabigatran	4 (1)	0 (0)	4 (2)	.31
Coumarin derivative	14 (4)	5 (4)	9 (3)	.77
No periprocedural OAC	7 (2)	1 (1)	6 (2)	.44

AAD *=* antiarrhythmic drug; AF *=* atrial fibrillation; AR *=* antiarrhythmic; BMI *=* body mass index; CAD *=* coronary artery disease; GFR *=* glomerular filtration rate; IQR = interquartile range; LA *=* left atrium; LDL *=* low-density lipoprotein; LAVI *=* left atrial volume index; LVEF *=* left ventricular ejection fraction; NOAC *=* novel oral anticoagulant; NT-proBNP *=* N-terminal prohormone of brain natriuretic peptide; OAC *=* oral anticoagulants; s/p = status post; TIA: transient ischemic attack.

∗ Statistically significant.

### Procedural data

#### Periprocedural hemodynamic parameters

##### Oxygen saturation

**Figure 2:**
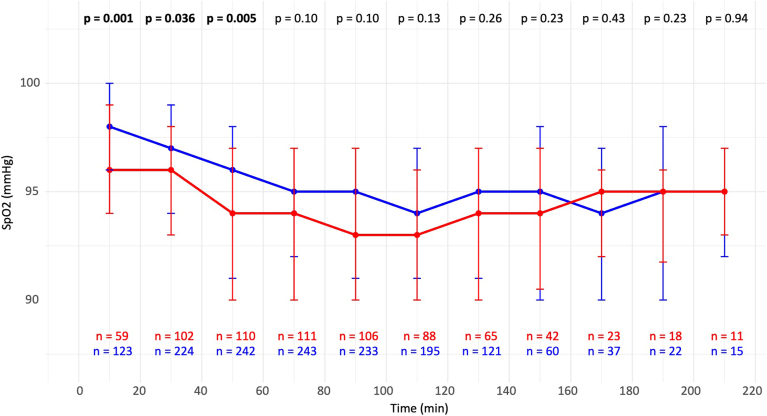
SpO_2_ levels during the procedure in both groups. SpO_2_ = oxygen saturation.

A total of 254 patients (67%) experienced at least 1 episode of hypoxia (SpO_2_ of <90%) with no difference in incidence between groups (*P* = .8). However, among those who experienced hypoxia, obese patients had a higher number of episodes (median 4 vs 2, *P* < .05). SpO_2_ was assessed using median values over the consecutive 20-minute intervals. Obese patients had significantly lower SpO_2_ levels at baseline (96% vs 98%, *P* = .001) and 30 minutes (96% vs 97%, *P* = .04) and 50 minutes of the procedure (94% vs 96%, *P* = .005) (Figure 2A). At later time points, no significant differences were observed. Importantly, median SpO_2_ values and the first quartile remained above 90% in both groups during the entire procedure. The intraprocedural oxygen saturation drop was not different between groups (Figure 2B). No patient required mechanical ventilatory support during the procedure (eg, bag-mask ventilation, noninvasive positive pressure ventilation, or intubation).

##### BP

**Figure 3:**
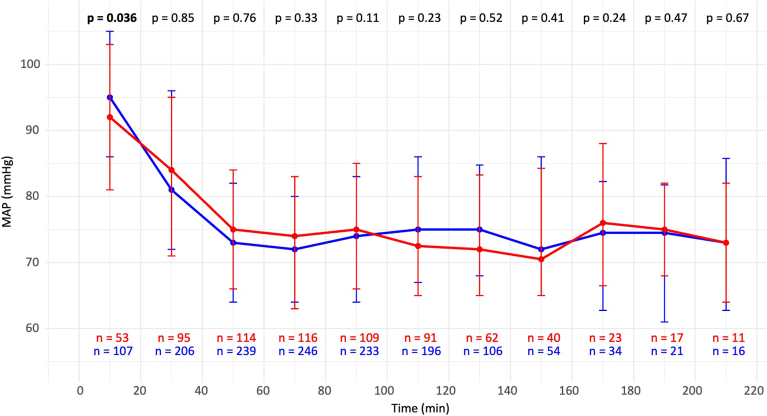
MAP levels during the procedure in both groups. MAP = mean arterial pressure.

Baseline MAP was slightly lower in obese patients (92 vs 95 mm Hg, *P* = .04). Overall, 250 patients (66%) experienced at least 1 episode of hypotension, with a comparable incidence in both groups (63% vs 67%, *P* = .4). The number of hypotensive episodes did not significantly differ between obese and nonobese patients. MAP was assessed using median values over consecutive 20-minute intervals. A sedation-induced drop in MAP was observed, with no significant difference in MAP reduction between groups, regardless of the ablation technique (Figure 3). There was a tendency toward lower MAP levels in obese patients after 100 minutes of the procedure, although the differences were not statistically significant. No cases required vasopressor administration or discontinuation of continuous propofol infusion.

**Figure 4:**
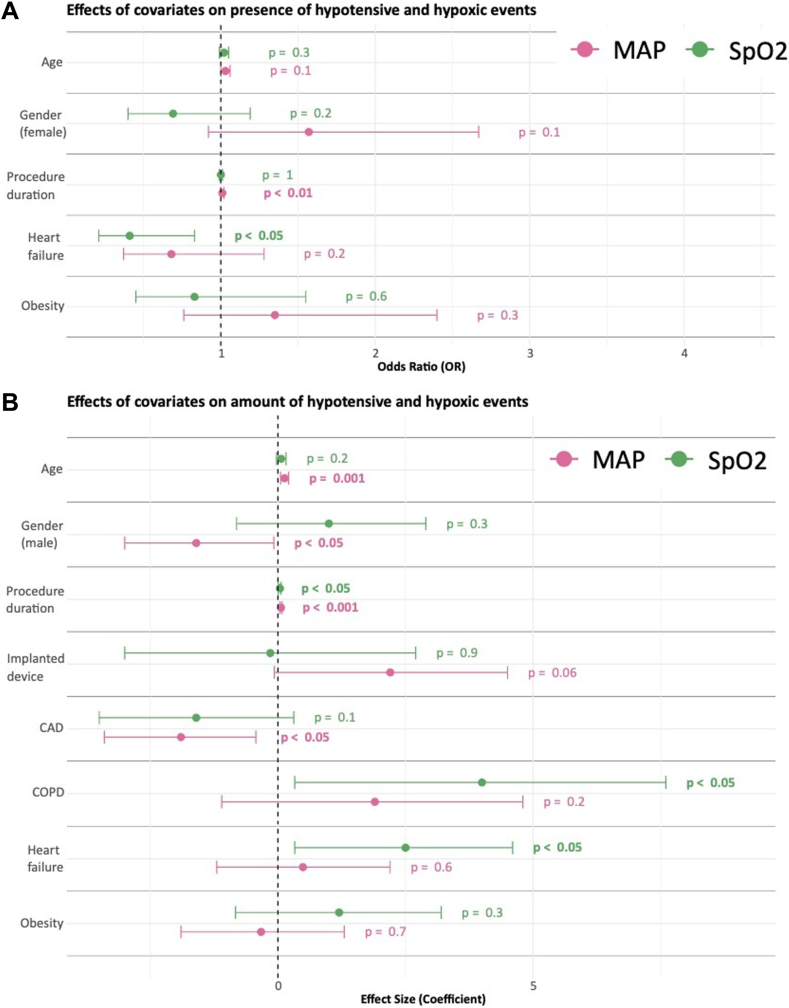
**A:** Effects of covariates on the presence of hypotensive and hypoxic events. **B:** Effects of covariates on the number of hypotensive and hypoxic events. CAD = coronary artery disease; COPD = chronic obstructive pulmonary disease; MAP = mean arterial pressure.

Logistic regression was performed to analyze the association between obesity and hemodynamic compromise, and linear regression was used to assess the number of hypotensive and hypoxic events (Figure 4). Obesity alone was neither a risk factor for hypoxia nor for hypotension.

In the regression analysis, a reduced LVEF (<35%) emerged as a major risk factor for both the presence and number of hypoxia episodes (*P* < .05 for both). Longer procedure duration (*P* < .05) and chronic obstructive pulmonary disease (COPD) (*P* < .05) were associated with a higher number of hypoxia episodes.

The major risk factor for both the presence (*P* < .01) and number of hypotension episodes (*P* < .001) was longer procedure duration. In addition, an increased number of hypotension episodes was associated with older age (*P* = .001) and female gender (*P* < .05).

#### Ablation and radiation parameters

The procedural ablation and radiation parameters are presented in Table 2. Of the 381 procedures performed, 297 (78%) were conducted with single-shot devices, 266 (69%) by CB ablation, and 36 (9%) by PFA. The remaining 84 procedures (22%) were performed by RF ablation (Figure 1B). Ablation success, defined as isolation of all pulmonary veins, was achieved in all procedures (381 of 381). In obese patients, procedure duration was significantly longer for all ablation techniques (76 vs 71 minutes, *P* = .012) and in RF ablation (144 vs 97 minutes, *P* = .016), but without differences for single-shot techniques (Supplementary File 1). Although the time from the start of procedure to TSP was not different among groups, LA dwell time was longer in obese patients in all procedures (60 vs 52 minutes, *P* < .001) and both single-shot device procedures (52 vs 50 minutes, *P* = .04) and RF ablations (120 vs 76 minutes, *P* = .01). Fluoroscopy time was comparable for obese and nonobese patients (*P* = .47), whereas fluoroscopy dose was higher in the obese group (1063 vs 402 cGy × cm^2^, *P* < .01) in total and for both single-shot device and RF techniques (Supplemental File 1). Obese patients also required a higher dose of fentanyl in total and for single-shot procedures (0.1 vs 0.07 mg, *P* < .01) and a higher dose of contrast medium (80 vs 70 mL, *P* = .061) for single-shot procedures only. Both fentanyl and contrast medium doses were comparable for RF ablations.

**Table 2:** Procedural ablation data

Procedural data	Total (n = 381)	BMI of ≥30 kg/m^2^ (n = 120)	BMI of <30 kg/m^2^ (n = 261)	*P* value
Ablation strategy, %				
Single-shot device	297 (78)	86 (72)	211 (81)	.045∗
CBA	261 (69)	76 (63)	185 (71)	.14
PFA	36 (9)	10 (8)	26 (10)	.61
RF	84 (22)	34 (28)	50 (19)	<.001∗
Procedure duration, min				
Total	72 [59–94]	76 [62–109]	71 [57–87]	.012∗
Single-shot device	68 [56–81]	71 [57–81]	67 [55–81]	.39
RF	109 [78–162]	144 [93–171]	97 [72–132]	.016∗
Preparation time (femoral puncture to TSP), min				
Total	17 [10–24]	17 [11–23]	17 [10–24]	.94
Single-shot device	17 [11–23]	17 [10–22]	17 [11–23]	.74
RF	16 [10–25]	18 [12–27]	15 [9–25]	.6
LA dwelling time (TSP to end of procedure), min				
total	54 [44–72]	60 [48–89]	52 [42–69]	<.001∗
Single-shot device	51 [41–62]	53 [47–66]	50 [41–61]	.04∗
RF	91 [61–138]	121 [74–150]	76 [55–113]	.01∗
Fluoroscopy time, min				
Total	13 [8–18]	13 [8–19]	12 [7–18]	.47
Single-shot device	13 [8–18]	13 [8–19]	13 [8–18]	.58
RF	9 [3–17]	10 [5–20]	9 [2–16]	.37
Radiation dose, cGy × cm^2^				
Total	523 [242–1192]	1063 [496–2356]	402 [212–803]	<.001∗
Single-shot device	509 [247–996]	939 [480–1633]	395 [215–739]	.001∗
RF	945 [220–2325]	2104 [814–3766]	412 [172–1480]	<.001∗
Dose of contrast medium, mL				
Total	75 [50–100]	80 [50–100]	70 [50–100]	.06
Single-shot device	80 [60–100]	90 [60–110]	80 [60–100]	<.001∗
RF	50 [35–80]	54 [43–80]	50 [30–70]	.21
Dose of fentanyl, mg				
Total	0.1 [0.05–0.1]	0.1 [0.05–0.1]	0.1 [0.05–0.1]	<.001∗
Single-shot device	0.1 [0.05–0.1]	0.1 [0.05–0.1]	0.07 [0.05–0.1]	<.001∗
RF	0.1 [0.1–0,15]	0.1 [0.05–0.15]	0.1 [0.1–0.1]	.18

BMI = body mass index; CBA = cryoballoon ablation; CTI = cavotricuspid isthmus; LA = left atrium; PFA = pulsed field ablation; RF = radiofrequency ablation; TSP = transseptal puncture.

∗ Statistically significant.

### Long-term ablation outcomes

**Figure 5:**
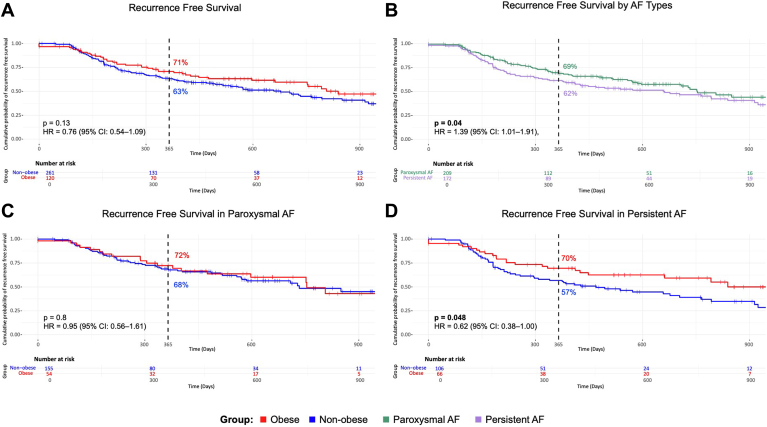
Kaplan-Meier analysis. **A:** Comparison of AF recurrence-free survival of obese vs nonobese patients. **B:** Comparison between paroxysmal and persistent AF. **C:** Comparison of obese vs nonobese patients with paroxysmal AF. **D:** Comparison of obese vs nonobese patients with persistent AF. AF = atrial fibrillation.

The follow-up data could be collected from 335 patients (88%) with a follow-up duration of 455 days [184–686]. The overall freedom from AF after 1 year was 66%. For paroxysmal AF, it was higher than for persistent AF (69% vs 62%, *P* = .043) (Figure 5B). The Kaplan-Meier analysis is shown in Figure 5.

The 1-year freedom from AF was not significantly different between obese and nonobese patients (71% vs 63%, *P* = .13) (Figure 5A). The subanalysis of the effect of BMI on the long-term success rate of ablation for 1 year, based on the AF type, is shown in Figure 5C and 5D. For patients with paroxysmal AF, the Kaplan-Meier analysis showed similar recurrence-free survival after 1 year, 72% for obese and 68% for nonobese patients (*P* = .8). However, in patients with persistent AF, obese patients had a higher 1-year recurrence-free survival than nonobese patients (70% vs 57%, *P* = .048). Comparison of baseline characteristics between obese and nonobese patients with persistent AF showed that nonobese patients were older, had a larger LA, and had a higher N-terminal prohormone of brain natriuretic peptide level (Supplementary File 2). There were no significant differences in 1-year recurrence rates among the obesity subgroups (BMI of 30–35, 35–40, and >40 kg/m^2^) (Supplemental File 3).

Eight patients (2%), 5 obese and 3 nonobese, experienced recurrence during the 3-month blanking period. At follow-up, 339 patients (89%) were on beta-blocker therapy, 5 (1.3%) on amiodarone, and 15 (4%) on class I antiarrhythmic drugs.

### Complications and in-hospital management

The overall complication rate was 3.4% (Table 3), with no impact of BMI on procedural safety (3.3% vs 3.4%, *P* = 1). In 1 patient from the nonobese group, cardiac perforation leading to fulminant tamponade and death occurred. There were no other cases of cardiac tamponade. Seven patients (1.8%) experienced phrenic nerve injury, which was persistent at discharge, 3 (2.5%) in the obese and 4 (1.5%) in the nonobese group. All of them showed total recovery in a 6-month to 1-year follow-up. In 3 patients (0.7%), cerebrovascular ischemic thromboembolic events occurred, 1 in the obese group and 2 in the nonobese group: 2 transient ischemic attacks and 1 stroke owing to arteria media cerebri occlusion resolved by immediate thrombus aspiration. All 3 patients were free of neurologic deficits at discharge. Two patients from the nonobese group experienced access site complications. No periprocedural intubation, esophageal complications, or procedure-related major bleeding was recorded. No significant differences in periprocedural complications were observed among the obesity subgroups (BMI of 30–35, 35–40, and >40 kg/m^2^) (Supplemental File 4). The median postprocedural hospital stay was 1 night, without difference between the obese and nonobese groups (*P* = .2).

**Table 3:** Periprocedural complications and in-hospital management

Procedural complications	Total (n = 381)	BMI of ≥30 kg/m^2^ (n = 120)	BMI of <30 kg/m^2^ (n = 261)	*P* value
Complications, %				
Total	13 (3.4)	4 (3.3)	9 (3.4)	1
Cardiac perforation leading to death	1 (0.3)	0 (0)	1 (0.38)	1
Phrenic nerve palsy	7 (1.8)	3 (3)	4 (2)	.68
Femoral access site complications, including bleeding that required transfusion	2 (0.5)	0 (0)	2 (0.7)	1
Stroke or transient ischemic attack	3 (0.7)	1 (0.8)	2 (0.7)	1
Intubation	0	0	0	
Esophageal complication	0	0	0	
Length of stay, d (median, IQR)	1 [1–1]	1 [1–1]	1 [1–1]	.23

BMI = body mass index; IQR = interquartile range.

## Discussion

The present study was a prospective observational comparison of the safety and efficacy of de novo PVI between obese and nonobese patients with a particular focus on periprocedural sedation and hemodynamic parameters.

### Patient population

Obese patients in our study were younger and had a higher burden of comorbidities. This aligns with the current evidence that obesity is an established risk factor for AF and obese individuals tend to develop AF at a younger age.^9^^,^^10^^,^^14^^,^^15^ In addition, the higher prevalence of comorbidities in the obese group reflects the well-known association between obesity and cardiovascular disease.^16^, ^17^, ^18^ Consistent findings are reported in other studies, comparing CA between obese and nonobese patients.^19^, ^20^, ^21^, ^22^, ^23^, ^24^, ^25^, ^26^, ^27^ Higher prevalence of persistent AF in obese patients may be explained by the lower success rates of cardioversion in this group.^28^^,^^29^ Both groups did not differ in other factors that may affect the sedation complexity, such as chronic lung disease, smoking, or alcohol abuse.

### PVI under DS in obese patients

DS is an established periprocedural anesthesia technique for PVI, with propofol and midazolam as the most frequently used hypnotic agents and remifentanil and fentanyl as the most widely administered opioids.^11^ Data on the impact of the sedation type on long-term PVI outcomes vary across studies.^30^^,^^31^ The choice of anesthesia depends on the operator’s preference, patient characteristics, and legal regulations in different countries; current guidelines offer no specific recommendations on this issue.^32^^,^^33^ In our department, all PVI procedures are performed under DS, supervised by an experienced cardiologist and a well-trained nurse, in line with standard practice in Germany.^34^^,^^35^

The main challenges of using DS in obese patients are (1) the impact of comorbidities on sedation and (2) challenges in drug dose adjustment.

(1) As BMI increases, lung volume exponentially decreases; moreover, obese patients often experience ventilation-perfusion mismatch.^36^ During DS, obstructive sleep apnea, airway collapse, and obstruction are frequently detected, complicating the airway management.^37^^,^^38^
(2) In obese patients, pharmacokinetics and pharmacodynamics are affected owing to increased cardiac output, lean body weight, fat mass, and extracellular fluid volume.^39^ This leads to a more rapid onset of drug effects and accumulation of lipophilic propofol in fatty tissue compartments, which may lead to hypotension, challenges in dose adjustment, and prolonged drug elimination.^34^^,^^40^, ^41^, ^42^

These concerns may result in scheduling obese patients for procedures under GA or even in refusing PVI owing to anticipated procedure complexity.

### Periprocedural respiratory and hemodynamic parameters

The feasibility of PVI in obese patients under DS using different ablation techniques has been previously reported; however, these studies lacked a detailed analysis of periprocedural respiratory and hemodynamic parameters and their comparison with those of nonobese patients.^24^^,^^43^, ^44^, ^45^, ^46^, ^47^

With the detailed monitoring of the periprocedural respiratory parameters, we found that maintaining adequate respiratory parameters was only slightly more complex in obese patients, because they experienced more hypoxia episodes (SpO_2_ of <90%) than nonobese individuals, but without the need for noninvasive positive pressure ventilation or intubation. Despite some lower median SpO_2_ values in this group, the difference remained clinically irrelevant, given that both the median and the first quartile values stayed above 90%. Multivariate analysis showed that not obesity but reduced LVEF, longer procedure duration, and COPD were associated with a higher risk of hypoxia in the study cohort. The increased hypoxia risk in patients with COPD may be explained with a higher risk of bronchospasm and laryngospasm (according to known data from GA), in patients with reduced LVEF by a tendency toward volume overload and in longer procedures by propofol accumulation and by the simple fact that a longer procedure increases the overall window of time during which hypoxic episode can occur.^48^

Aside from a slightly lower baseline MAP in obese patients, no differences in the presence and number of hypotensive episodes or in MAP reduction were observed between groups. The tendency toward lower BP after 100 minutes of the procedure in obese individuals may be associated with greater propofol accumulation, although that was not statistically significant. In multivariate analysis, longer procedure duration, older age, and female gender were identified as risk factors for hypotension. Hypotension after the administration of propofol and fentanyl is a known adverse effect in elderly patients; a longer procedure duration, owing to the same reasons, explained for the respiratory parameters.^49^ A protective effect of coronary artery disease against hypotension may be explained by the higher concomitant incidence of hypertension in these patients.

Thus, when anticipating the complexity of DS during PVI, not only obesity but also the other identified risk factors should be considered. Although closer and more intensive airway monitoring may be required, BP management seems to be comparable between groups.

### Ablation parameters

Prolonged procedure time in obese patients—mainly driven by RF ablations—and prolonged LA dwell time overall and across all techniques could be explained by a larger LA size and a higher number of desaturation episodes in this group. Only a few previous studies reported periprocedural parameters. Procedure time was described as both unchanged and prolonged in the obese group; LA dwell time was evaluated in only 1 study and was also found to be prolonged; all papers reported a higher radiation dose and a higher dose of contrast medium, probably owing to poorer visibility of cardiac structures.^23^^,^^24^^,^^26^^,^^43^^,^^45^, ^46^, ^47^ Similar contrast dye dose with the RF technique is probably caused by the ability to visualize the veins when using the 3-dimensional mapping, and therefore, RF ablation could be the technique of choice in obese patients with impaired renal function.

### Safety

The safety profile was comparable between obese and nonobese patients, with no significant difference in complication rate. Notably, the vascular access site complications also did not differ between groups. Although the exact duration of the femoral puncture was not documented, the so-called “preparation time,” defined as the interval from the initiation of local anesthesia to TSP, was similar in both groups. This interval may serve as a surrogate for the femoral puncture complexity. Some previous studies have reported higher rates of femoral access complications in obese patients.^45^^,^^50^ We believe that ultrasound-guided puncture, which was performed in all patients of our study, may have mitigated the risk. Ultrasound-guidance showed a significant decrease in the vascular complication rate compared with an anatomic-landmark-based approach.^51^^,^^52^

### Long-term success

In our study, patients with a BMI >30 kg/m^2^ had a similar recurrence rate to those with a normal BMI. Interestingly, in a subgroup analysis of patients with persistent AF, obese patients had a lower recurrence rate (70% vs 57%). This finding could be explained by the larger LA volume and higher N-terminal prohormone of brain natriuretic peptide levels observed in nonobese patients, possibly indicating a more advanced stage of AF and underlying atrial cardiomyopathy. The impact of BMI on long-term ablation outcomes was analyzed in various studies, with mixed findings, given that the BMI threshold associated with poorer long-term prognosis varies between 30 and 35 kg/m^2^. For CB, some studies report no effect of obesity on long-term success,^27^^,^^43^ whereas others have shown reduced 1-year success rate in severely (BMI of >35 kg/m^2^) and “very severely” obese patients (BMI of >40 kg/m^2^).^45^^,^^46^ In patients ablated with the RF technique, 1 study reported a negative impact of obesity on prognosis when a BMI exceeded 35 kg/m^2^,^25^ whereas another observed worse outcomes already at a BMI of >30 kg/m^2^.^23^ There is no preferred ablation technique in obese patients, given that only a few retrospective studies reported a comparison among different ablation techniques in obese patients; one of them showed superior recurrence outcomes of PFA to CB PVI,^44^ and another study showed comparable recurrence rate between RF and CB PVI.^47^

Therefore, when a BMI exceeds 35 kg/m^2^, outcomes are worse. However, in patients with a BMI between 30 and 35 kg/m^2^, findings are mixed. These patients may benefit from PVI to a similar extent and with a comparable safety profile as nonobese patients, which aligns with our findings.

Although weight reduction in obese patients is known to decrease AF burden,^53^^,^^54^ physical exercise can be hampered by the arrhythmia itself, constituting a vicious circle for obese patients. Moreover, postponing the ablation procedure may reduce its success, given that early PVI in paroxysmal AF has been shown to improve rhythm control efficacy and outcomes.^55^^,^^56^

### Limitations

This was a single-center, nonrandomized prospective study, and therefore, a selection bias cannot be entirely excluded. Obese patients represented approximately one-third of the cohort, and the distribution of ablation techniques was not uniform with the predominance of CB ablation. Moreover, patients requiring additional ablation beyond PVI were excluded, which may limit the applicability of these data to real-world patients with AF. Follow-up data were not available for all enrolled patients, owing to some being lost to follow-up. In addition, most follow-up assessments relied on 24-hour Holter ECG or symptomatic AF recurrence, rather than continuous monitoring by implantable devices (eg, implantable loop recorder); therefore, underestimation of the recurrence rate is probable. However, this is true for both groups and therefore should not lead to bias in the comparison.

## Conclusion

Obesity was not an independent risk factor for periprocedural hypoxia or hypotension and did not affect safety or long-term success. Obesity alone should not be considered a reason to exclude patients from undergoing PVI under DS.

## Figures and Tables

**Supplementary Figure 1:**
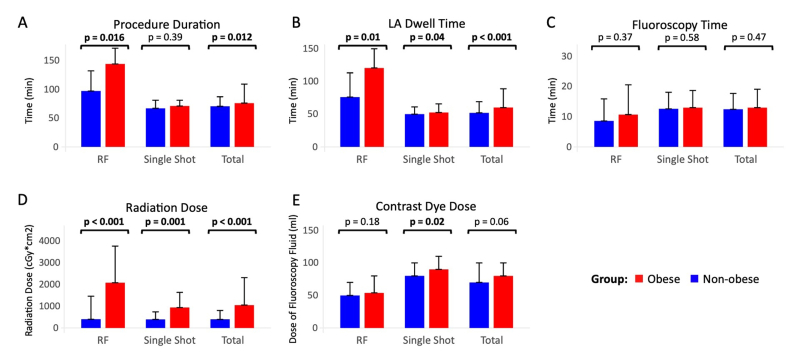


**Supplementary Figure 2:**