# Supplementary material for: De novo pulmonary vein isolation in obese vs nonobese patients under deep sedation: Does obesity increase procedure complexity?
Source: Heart Rhythm O2. 2025 Jul 8;6(10):1524–35. doi: 10.1016/j.hroo.2025.06.024 (PMC12570196; doi:10.1016/j.hroo.2025.06.024)
Supplement: Supplementary Material [file mmc1.docx]

**Supplementary file 2.**

Baseline characteristics in patients with persistent AF according to BMI

|  | Total  (n=172) | BMI ≥ 30 kg/$\boldsymbol{m}^{\boldsymbol{2}}$ (n=66) | BMI < 30 kg/$\boldsymbol{m}^{\boldsymbol{2}}$ (n=106) | p-value |
| --- | --- | --- | --- | --- |
| Demographics |  |  |  |  |
| Age, yrs | 69 [61; 75] | 63 [58; 72] | 71 [64; 76] | **< 0.001** |
| Male, % | 115 (67) | 43 (65) | 72 (68) | 0.71 |
| AF |  |  |  |  |
| Time after initial diagnosis |  |  |  |  |
| < 6 M | 44 (26) | 15 (23) | 29 (27) | 0.50 |
| ≥ 6 M | 86 (50) | 35 (53) | 51 (48) | 0.53 |
| CHA_2_DS_2_-VA-Score |  |  |  |  |
| Median, IQR | 3 [2; 4] | 3 [2; 4] | 3 [2; 4] | 0.09 |
| ≥ 2, % | 146 (85) | 51 (77) | 95 (90) | **0.03** |
| Comorbid conditions |  |  |  |  |
| BMI, kg/m^2^ | 28 [24; 32] | 34 [32; 38] | 25 [22; 28] | **0** |
| Hypertension, % | 139 (81) | 59 (89) | 80 (75) | **0.02** |
| Diabetes mellitus, % | 33 (19) | 15 (23) | 18 (17) | 0.35 |
| CAD, % | 73 (42) | 26 (40) | 47 (44) | 0.52 |
| Impaired kidney function (GFR < 60), % | 59 (34) | 20 (30) | 39 (37) | 0.38 |
| s/p stroke/TIA, % | 15 (9) | 2 (3) | 13 (12) | **0.05** |
| Respiratory disease, % | 20 (12) | 10 (15) | 10 (9) | 0.33 |
| Smoker, % | 61 (35) | 27 (41) | 34 (32) | 0.24 |
| Alcohol abuse, % | 9 (5) | 4 (6) | 5 (5) | 0.73 |
| Implanted device, % | 20 (12) | 5 (8) | 15 (14) | 0.23 |
| Laboratory values |  |  |  |  |
| LDL, mmol/l | 3 [2; 3] | 3 [2; 4] | 2 [2; 3] | 0.15 |
| NT-proBNP, ng/l | 1162 [539; 2179] | 892 [401; 1254] | 1423 [788; 2821] | **< 0.001** |
| Glucose, mg/dl | 100 [91; 114] | 103 [91; 129] | 99 [90; 110] | 0.09 |
| Echocardiographic data |  |  |  |  |
| LVEF, % | 54 [42; 60] | 55 [44; 60] | 51 [40; 60] | 0.91 |
| LVEF < 35%, % | 27 (16) | 7 (11) | 20 (19) | 0.20 |
| LA volume, ml | 87 [70; 100] | 88 [65; 105] | 86 [70; 98] | 0.62 |
| LAVI ml/m^2^ | 42 [33; 49] | 38 [29; 43] | 45 [38; 51] | **0.02** |
| AAD therapy, % |  |  |  |  |
| Beta blockers | 161 (94) | 61 (92) | 100 (94) | 0.75 |
| Amiodaron | 17 (10) | 9 (14) | 8 (8) | 0.20 |
| Class I AR | 6 (3) | 2 (3) | 4 (4) | 1 |
| OAC therapy, % |  |  |  |  |
| NOACs | 161 (94) | 62 (94) | 99 (93) | 1 |
| Apixaban | 82 (48) | 37 (56) | 45 (42) | 0.08 |
| Rivaroxaban | 59 (34) | 20 (30) | 39 (37) | 0.38 |
| Edoxaban | 18 (10) | 5 (8) | 13 (12) | 0.44 |
| Dabigatran | 2 (1) | 0 (0) | 2 (2) | 0.52 |
| Coumarin derivative | 9 (5) | 4 (6) | 5 (5) | 0.73 |
| No periprocedural OAC | 2 (1) | 0 (0) | 2 (2) | 0.52 |

Abbreviations as in Table 1
