# Supplementary material for: De novo pulmonary vein isolation in obese vs nonobese patients under deep sedation: Does obesity increase procedure complexity?
Source: Heart Rhythm O2. 2025 Jul 8;6(10):1524–35. doi: 10.1016/j.hroo.2025.06.024 (PMC12570196; doi:10.1016/j.hroo.2025.06.024)
Supplement: Supplementary Material [file mmc2.docx]

**Supplementary file 4.**

Periprocedural complications and in obesity subgroups

|  | Total (n=120) | BMI = 30-35 kg/m^2^ (n=78) | BMI = 35-40 kg/m^2^ (n=23) | BMI > 40 kg/m^2^ (n=19) | p-value |
| --- | --- | --- | --- | --- | --- |
| hypoxia (SpO2 < 90%) |  |  |  |  |  |
| hypoxic event, % | 81 (68) | 52 (67) | 16 (70) | 13 (68) | 0.96 |
| number of hypoxic events | 4 [0; 9] | 4 [0; 9] | 3 [1; 8] | 5 [0; 13] | 0.95 |
| hypotension (SBP < 90 mmHg) |  |  |  |  |  |
| hypotensive event, % | 82 (68) | 56 (72) | 16 (70) | 10 (53) | 0.27 |
| number of hypotensive events | 3 [0; 9] | 3 [0; 9] | 5 [0; 12] | 1 [0; 4] | 0.79 |
| hypotension (MAP < 65 mmHg) |  |  |  |  |  |
| hypotensive event, % | 75 (63) | 51 (65) | 15 (65) | 9 (47) | 0.33 |
| number of hypotensive events | 2 [0; 6] | 2 [0; 8] | 3 [0; 8] | 0 [0; 2] | 0.88 |
| Complications, % |  |  |  |  |  |
| Total | 4 (3) | 2 (3) | 2 (9) | 0 (0) | 0.28 |
| Phrenic nerve paralysis | 3 (3) | 1 (1) | 2 (9) | 0 (0) | 0.16 |
| Stroke/TIA | 1 (1) | 1 (1) | 0 (0) | 0 (0) | 1 |

BMI: body mass index; MAP: mean arterial pressure; SBP: systolic blood pressure; SpO2: peripheral oxygen saturation; TIA: transient ischemic attack
